# Thermal Characteristics of Expandable Graphite–Wood Particle Composites

**DOI:** 10.3390/ma13122732

**Published:** 2020-06-16

**Authors:** Kwanok Chun, Jeonggon Kim, Dongho Rie

**Affiliations:** 1 Graduate School of Safety Engineering, Incheon National University, Incheon 22012, Korea; kochun0915@hanmail.net; 2 Department of Advanced Materials Science and Engineering, Incheon National University, Incheon 22012, Korea; jyj309@inu.ac.kr; 3 Fire Disaster Prevention Research Center, Incheon National University, Incheon 22012, Korea

**Keywords:** expandable graphite composite, total heat release rate, flame retardancy, expansion rate, thermal conductivity, fine lattice layer structure

## Abstract

According to the Fire Statistics Yearbook of the National Fire Agency of the Republic of Korea, the total number of fires in 2018 was 42,338, which resulted in 2500 victims and amounted to property damages of approximately 560 billion KRW. The number of fires in buildings where wood was used as a finishing material was 28,013 (66%) in that period. To minimize human and property damage, composite materials were prepared by mixing wood particles with expandable graphite. The physical and thermal properties of the composite materials were investigated. It was observed that the expansion rate increased by 341.7% according to the expandable graphite content. Additionally, the total heat released and the thermal conductivity decreased from 38.63 to 2.5 MJ/m^2^ and from 24.62 to 7.8 W/m·K. The time to inactivity of white mouse in the smoke toxicity test was 14.9 min and exceeded the toxicity standard for flame retardant performance. The expandable graphite added to composite materials adopted worm-like shapes as a result of combustion, and it formed a fine lattice layer structure with 16–22 μm gaps that could reduce thermal conductivity. In addition, we can minimize the damage to people and property in the event of a fire.

## 1. Introduction

With the development of residential settings, wood has witnessed extensive application as a construction material. Several research and development activities have been undertaken to minimize the damage caused to wooden materials used for interior finishing. However, wood fires still occur frequently in residential facilities and industrial sites, and recently, there has been an increase in human losses and material damage caused by such wood fires.

To minimize the damage caused by wood fires, the use of heat and combustion resistant wood materials in the interior and exterior of buildings has been actively recommended. Recently, there has been much active research and development of composite materials that use flame retardant materials such as expandable graphite and carbon nanomaterials. However, problems remain unsolved in terms of economic feasibility and application before these materials can be manufactured and used.

Wood particles are combustible materials consisting of cellulose (40%–45%), hemicellulose (20%–25%), and lignin (20%–30%), and are known to ignite in the temperature range of 225–275 °C [1]. In particular, compared with other materials, wood or wood particles that are used as interior materials in several parts of buildings are more vulnerable to fire. Therefore, to decrease the fire vulnerability of wood, flame retardant performance is enhanced by adding flame retardant materials to wood particles [2,3].

The principle and the general production method for using expandable graphite as a flame-retardant material are as follows. First, the natural and pyrolytic graphite particles are oxidized by immersing them in a mixture of sulfuric acid and an oxidant such as nitric acid, potassium permanganate, hydrogen peroxide, or perchloric acid. Subsequently, the graphites are washed with water and dried. Finally, the oxidized graphites are expanded by combustion. In detail, when the oxidized graphites are heated, gas is generated from the acid and oxidant present in them. This gas widens the interlayer space between the graphites, thus resulting in a flame-retardant effect. Several combustion experiments using a cone calorimeter have found that the flame-retardant property of composite of wood and expandable graphite improved as the total heat release rate (HRR) decreased [4,5,6]. Specifically, composites of expandable graphite with various materials such as polypropylene, ground tire rubber, erythritol, epoxy resin, paraffin, polypropylene/wax blends, and high-density polyethylene exhibit an improved flame-retardant property [7,8,9].

Expandable graphite has several applications including flame retardant materials, lithium, medicines, heat generators, flame retardant plastics, and retardant polyurethane foams owing to its expandability, thermal insulation, lubricity, plasticity, chemical stability, forming ability, and confidentiality. Several studies have investigated the physical and thermal characteristics of expandable graphite. Krupa et al. studied the physical behaviors of composite phase change materials such as polyethylene and paraffin wax containing expandable graphite and reported that addition of expandable graphite decreased the reduction rate of composite materials [10]. Modesti added 5–25 wt.% expandable graphite to polyisocyanurate-polyurethane foams as a flame retardant, and found that the peak HRR decreased, while the CO/CO_2_ average value and oxygen index increased, which indicates improved thermal stability [11]. Kruger et al. conducted a study on the thermal behavior of flame-retardant polyethylene with expandable graphite and revealed that the expandable graphite formed char in the combustion layer, which interferes with the heat flow [12]. Wang et al. reported that the thermal conductivity of a polymer composite filled with expandable graphite sheet fillers increased when the expandable graphite layer grew thicker [13]. Furthermore, Zhang and Fang reported that the expanded graphite in paraffin-expandable graphite composite prevents the leakage of molten paraffin, stores heat, and plays the role of an auxiliary material that increases thermal conductivity [14]. Duan et al. found through microstructural analysis that an expandable graphite–calcium chloride hexahydrate composite exhibits increased thermal conductivity because of the high thermal conductivity of expandable graphite, and therefore, the composite could store thermal energy [15]. Choi et al. studied the thermal behavior of expandable graphite–erythritol composite and reported an increase in thermal conductivity as the expandable graphite content in the composite increased [16]. In addition, several studies have confirmed that composites exhibit increased heat transfer and heat storage capacities [17,18]. Moreover, studies on composite materials with expandable graphite demonstrated that the thermal conductivities of composites in the solid phase increase with the addition of expanded graphite [19]. Compared to previous studies, this study conducted experiments and analyzed the results based on two perspectives. First, the thermal properties of the composite with expandable graphite were compared with that of wood. Second, previous studies have reported the increasing trend of thermal conductivity in the solid state of the composite with expandable graphite. This study investigated the changes in the thermal conductivity of the composite following the expansion of expandable graphite after combustion. In this work, we investigated the physical properties, thermal characteristics, and structures of the composites containing expandable graphite, which expand when heated in fire. Through our findings, this study aimed to expand the applications of expandable graphite–wood particle composites.

## 2. Materials and Methods

### 2.1. Material Selection and Composite Fabrication

**Chart 1:**
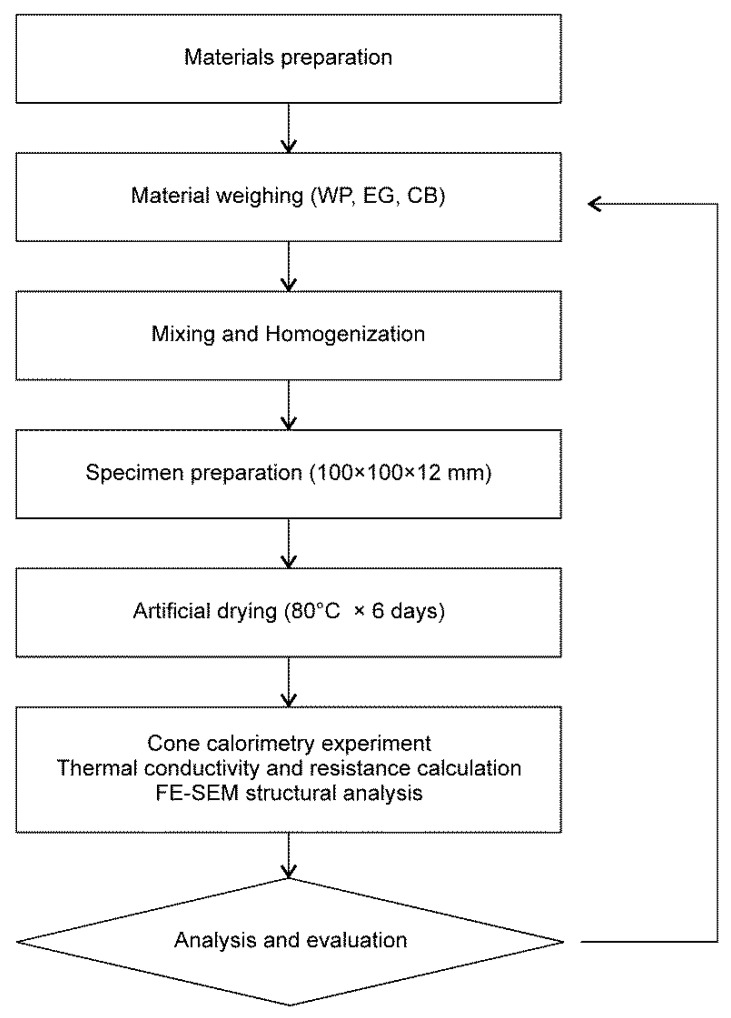
Flowchart of experimental sequence.

A flowchart representing the experiments performed in this study is provided in Chart 1. The experiment was carried out in the following order: Selection of materials for fabricating the expandable graphite composites; weighing, mixing and homogenization; specimen preparation; artificial drying; cone calorimetry experiment; and finally, data analysis and evaluation.

Wood particles (WP), expandable graphite (EG), and common binder (CB) were selected as the materials for experimentation. Table 1 presents the content, size, and morphology of WP and EG.

In general, woods are classified as either softwoods (e.g., Abies holophylla Maxim) or hardwoods (e.g., hard maple, oak). In detail, wood is an environmentally friendly material that is used as a construction subsidiary material and exhibits lower thermal conductivity, 0.12 W/m·K [20], as well as excellent machinability and heat resistance. However, it is combustible and vulnerable to fire, and suffers from warping or cracking caused by deformation. Wood particles are by-products of the wood shredding process and have similar chemical components to those of wood, which include natural polymers—cellulose, hemicellulose, lignin, and other extractives [21]. While the composition varies depending on the type of wood, woods primarily consist of cellulose ≤ 50%, hemicellulose ≤ 25%, and lignin ≤ 25%. C and H are readily combustible substances that are capable of undergoing continuous combustion even when the atmospheric oxygen level falls below 16%, owing to the additional O_2_ that is generated during the pyrolysis of cellulose [22]. The wood particles considered for this study were raw materials of domestically produced plywood and were generated during the processing of tropical hardwoods. The highest proportion of constituent elements of these wood particles were 55.61% carbon and 44.39% oxygen; moreover, their size was in the range of 80–100 mesh. The wood particles were dried in an artificial dryer at 80 °C for 72 h and were weighed repeatedly in 4 h intervals until there was no change in the mass of the specimen. The moisture content was calculated using Equation (1) [23], as follows:

MC (%) = [(Wm − Wd)/Wd] × 100,(1)

where MC is the moisture content (%), Wm is the weight of the sample before over drying, and Wd is the weight of the sample after over drying. The moisture content of the wood was measured to be 9.5%, 5.0%, and 2.0% after 24, 48, and 72 h of over drying, respectively.

Expandable graphite was procured from Qingdao Tianhe Graphite Co., Ltd., Qingdao, China. It contained 93.8% fixed carbon, 6.2% ash, and 0.68% moisture, and had the following properties; a pH of 4.5, expansion volume of 210 mL/g, expansion temperature of 180–200 °C, and particle size of +80 mesh (82%).

A 1:2 flour–water mixture that had been heated to 100 °C, prepared as a homogeneous liquid, and naturally cooled to room temperature for 24 h was used as the common binder for combining the expandable graphite and wood particles. The flour used as the major component of the common binder was medium flour that contained of 10%–13% of gluten, which is an insoluble protein complex.

Table 2 provides the mixing ratios of EG, WP, and CB for preparing composite samples.

Table 2 shows the ratios of the constituent materials in the composite material. Specimens were categorized into nine cases (from Case 1 to Case 9) based on the mixing conditions. For sample preparation, 100 g of wood particles and 300 g of binder were used as the basis, to which expandable graphite particles varying from 0–50 wt.% were added in increments of 5%–10%. Composites were fabricated by first loading wood particles and expandable graphite into a powder mixer and stirring it for 10 min. Later, the binder was added to the mixer, and the mixture was stirred for an additional 10 min. This mixture was transferred to a sample preparation mold with dimensions of 100 mm in length, 100 mm in width, and 12 mm in height and compressed under a force of 350 kgf for 5 min. A total of 27 specimens were prepared (three samples for each case) and dried in an artificial dryer at 80 °C for six days (144 h). The moisture contents of the wood-expandable graphite composites were measured using Equation (1). The initial weight (Wm) of the produced specimen was measured. After drying for 96 h in an artificial dryer, the weight (Wd) of the specimen was measured. After measuring the weight twice at intervals of 24 h, the weight change was less than 0.01 g, and the initial moisture content of 2.0% decreased to 0.02%, which was then used in the experiment. The combustion experiments were conducted three times for each specimen using a cone calorimeter, and the average value of the measured data was used for analysis.

**Figure 1:**
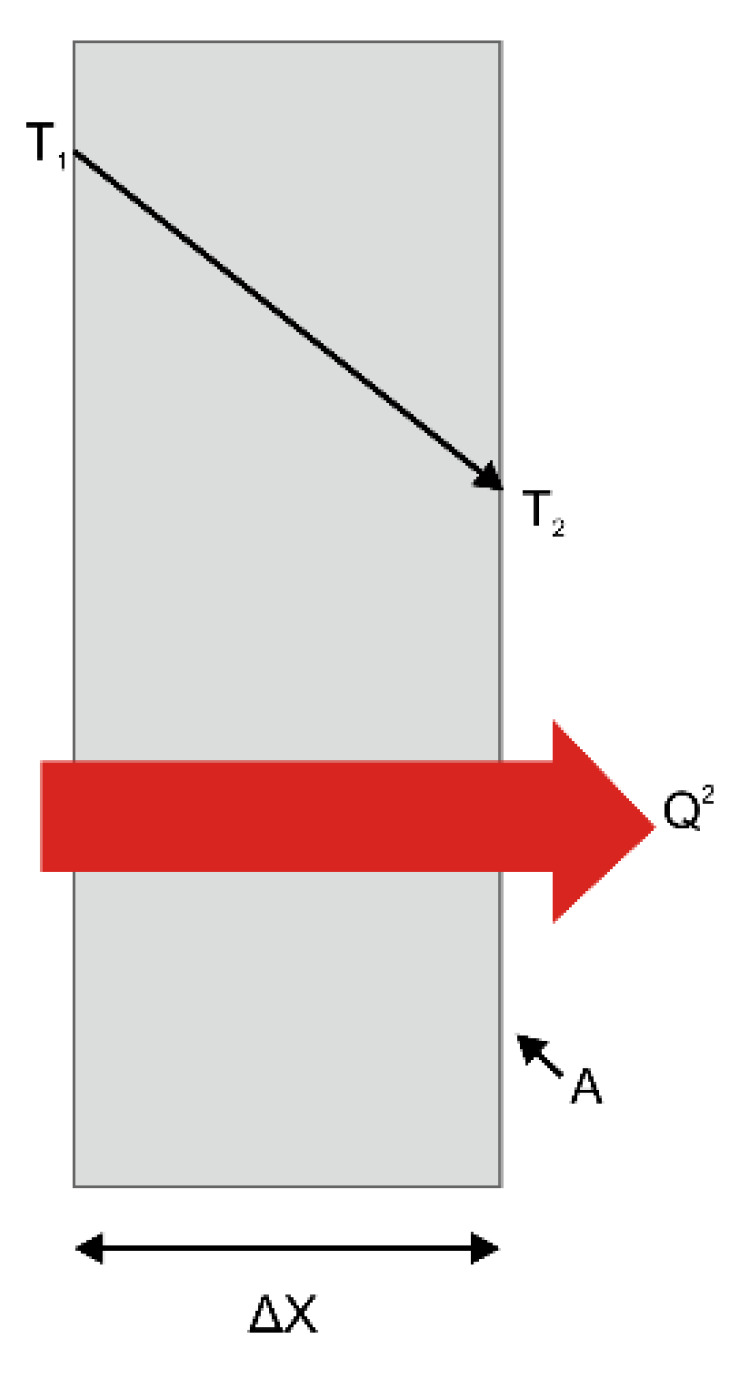
Heat conduction through a large plane of thickness Δx and area A.

Figure 1 depicts the heat transfer profile on a plane with a thickness, Δx, and a surface area, A. The temperature difference across the wall is ΔT = T_2_ − T_1_. According to the experiments, when ΔT or the A perpendicular to the heat transfer direction was doubled, the heat transfer (Q) doubled as well. However, when L was doubled, Q halved. Therefore, the heat transfer rate through the plane was found to be proportional to the temperature difference and the heat transfer area of the plane, and it was also found to be inversely proportional to the thickness of the plane.

Conduction heat transfer rate ∝ (area of plane) (temperature difference)/thickness of the plane

(2) $$Q_{\mathrm{COND}}=-\mathrm{kA}\frac{T_{1}-T_{2}}{\Delta x}=-\mathrm{kA}\frac{\Delta T}{\Delta x}$$

In the above equation, the proportionality constant, k, represents the thermal conductivity of the material, i.e., the capacity of the material to conduct heat. In the extreme case of Δx → 0, the above equation takes a differential form. In this case, Equation (2) is Fourier’s law of heat conduction, and dT/dx is the temperature gradient, i.e., the gradient of the temperature curve at position x in the T-x diagram (rate of change of T with x). This implies that the heat transfer rate is proportional to the temperature gradient in the heat transfer direction. When x increases, the temperature T_2_ decreases, and the temperature gradient becomes negative because heat is transferred to a lower temperature region. Therefore, a minus sign is added to Equation (2) so that the heat transfer value will turn positive for a heat flow in the positive x direction [24,25]. In other words, thermal conductivity can be defined as the amount of heat that flows through a material per unit time through a unit area with a temperature gradient of one degree per in unit distance. A material with a high thermal conductivity is an excellent thermal conductor, and a material with a low thermal conductivity is an insulator (heat insulator).

### 2.2. Cone Calorimetry Experiment

In the cone calorimetry experiment, the distance between the specimen and the cone heater of the cone calorimeter (FESTEC, Seoul, Korea) employed for the combustion experiment was fixed at 60 mm considering the expandability of expandable graphite. Moreover, the applied calorific value was set at 50 kW/m^2^. The experiment was performed for 5 min for each specimen at a laboratory temperature of 28 ± 1 °C and relative humidity (RH) of 60% ± 5%.

## 3. Results and Discussion

### 3.1. DSC and XRD Analyses

**Figure 2:**
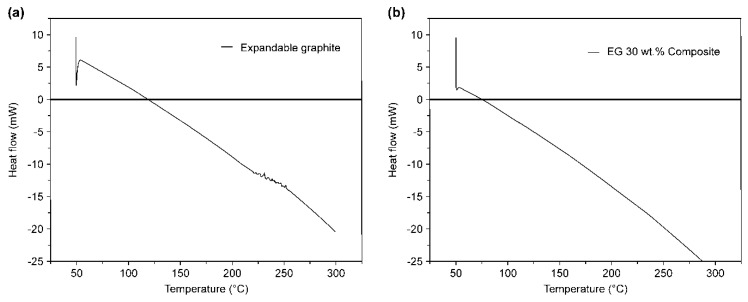
DSC analysis of the expandable graphite (**a**) and EG 30 wt.% composite (**b**).

Figure 2 shows the differential scanning calorimetry (DSC) analysis of the expandable graphite (a) and EG 30 wt.% composite specimen (b). The DSC analysis was conducted at a heating rate of 10 °C/min in an Ar Inert gas atmosphere. In Figure 2a, the expandable gas absorbed heat at approximately 54 °C, and the heat flow reached −20 mW at 300 °C. At this period, the heat absorption rate was 1.04 mW/min. In Figure 2b, the EG 30 wt.% composite absorbed heat at approximately 54 °C, and the heat flow reached −25 mW at 285 °C. At this period, the heat absorption rate was 1.174 mW/min. It was considered that the EG 30 wt.% composite had a faster heat absorption rate than that of the pure expandable graphite because it absorbed a large amount of heat to carbonize the wood particles in the specimen.

**Figure 3:**
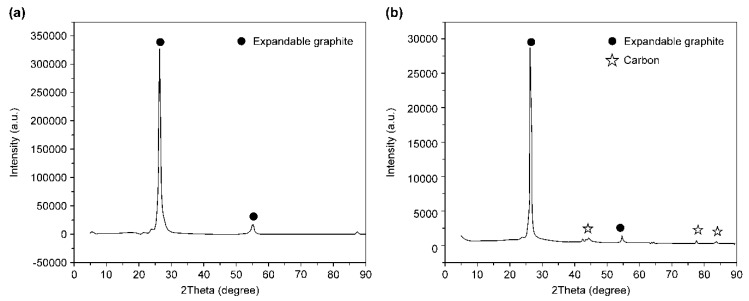
XRD analysis of the expandable graphite (**a**) before and (**b**) after combustion.

**Figure 4:**
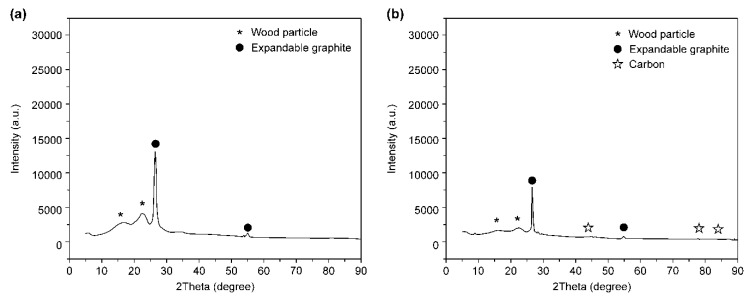
XRD analysis of the EG 30 wt.% composite specimen (**a**) before and (**b**) after combustion.

Figure 3 shows the X-ray diffraction (XRD) analysis before (a) and after (b) combustion of pure expandable graphite. In Figure 3a, the main peaks are at 27° and 55°, which were reflected by graphite. In Figure 3b, the peaks at 45°, 78°, and 83° confirmed that the expandable graphite was decomposed by heat to form carbon. Figure 4 shows the XRD analysis before (a) and after (b) combustion for the EG 30 wt.% composite. In Figure 4a, the diffraction peaks can be observed at 16° and 23° due to the effect of wood particles in the composite. Furthermore, Figure 4b shows that the diffraction peak of the wood particles lowered after the combustion of the sample, and that carbon was detected as a result of the combustion of the wood particles and thermal decomposition of expandable graphite.

### 3.2. Physical Characteristics of the Expandable Graphite Composites

**Figure 5:**
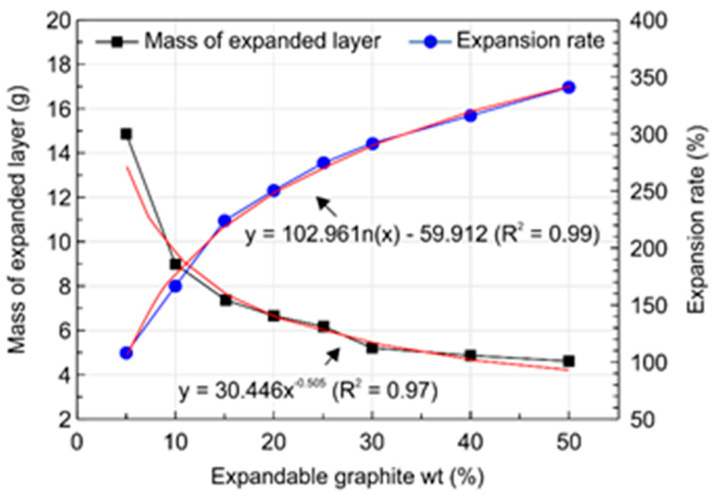
Expansion rate with expandable graphite content.

**Figure 6:**
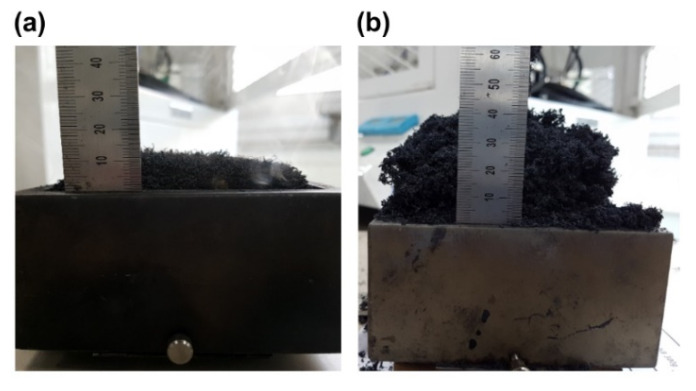
Images of expanded layer (**a**) EG 5 wt.% and (**b**) EG 50 wt.%.

Figure 5 shows the expansion rate based on the expandable graphite content. As the content of expandable graphite increased from 0 to 50 wt.%, based on the nine cases in Table 1, we observed that the expanded layer thickness increased from 0 mm to 13, 20, 27, 30, 33, 35, 38, and 41 mm. The expansion rates based on the initial specimen thickness of 12 mm before the experiment were 0.0%, 108.3%, 166.7%, 225.0%, 250.0%, 275.0%, 291.7%, 316.7%, and 341.7% (standard deviation: 78.2), respectively. In addition, Figure 6 displays the images of specimens with expandable graphite content of 0 and 50 wt.% after combustion and details the noticeable increase in the thickness of the expanded graphite layer due to heat. Such observation was shown to result from the wide expandability in the interlayers of expandable graphite that arises when the gas generated is exposed to heat.

**Figure 7:**
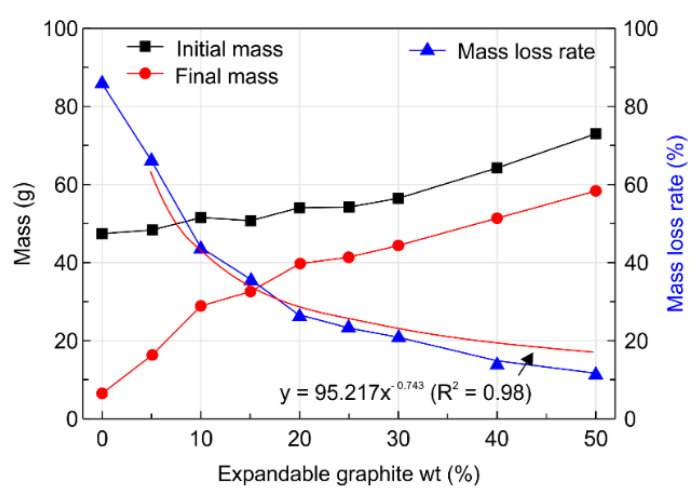
Mass loss rate variations with expandable graphite content.

Figure 7 details the mass loss rate of the specimen after the experiment according to the expandable graphite content. The mass loss rate was determined from the difference between the specimen masses before and after the experiment. The mass loss rates were 86.2%, 67.5%, 43.5%, 35.4%, 26.7%, 23.6%, 21.1%, 15.1%, and 12.4% (with a standard deviation of 25.1) for wood particle contents of 0, 5, 10, 15, 20, 25, 30, 40, and 50 wt.%, respectively, with expandable graphite of 0–50 wt.% by 5 wt.%, respectively. The mass loss rate gradually decreased as the expandable graphite content increased. The thickness of the expanded graphite layer (Leg) increased as the content of expandable graphite increased. The heat transfer became more disturbed as the expanded graphite layer thickness increased because the space formed between the expanded graphite interferes with heat transfer.

**Figure 8:**
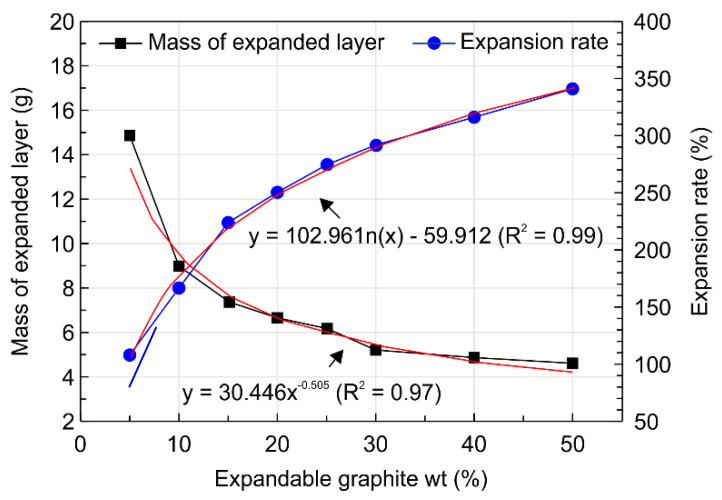
Variations in the expansion rate and mass of expanded layer with expandable graphite content.

The expansion rate and the mass of the expanded layer are graphed in Figure 8. As shown, the expansion rate increased to a maximum of 341.7% with the increase in expandable graphite content. Moreover, the mass of the expanded layer decreased from 19.25 to 4.52 g (standard deviation: 3.36) when the expandable graphite content increased from 0 to 50 wt.%. This seems to have been caused by the disturbance in heat transfer due to the increase in the thickness of the expanded layer, which interfered with the progress of combustion towards the bottom layer of the specimen.

**Figure 9:**
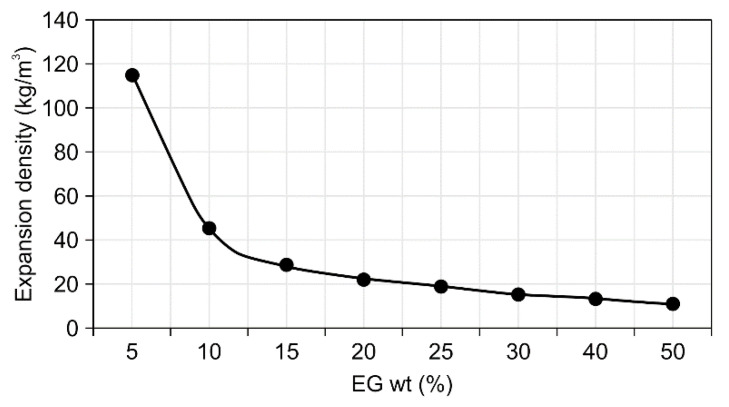
Variation in expanded graphite density with the expandable graphite content.

Figure 9 depicts the variation of expandable graphite density, which is determined by the thickness of the expansion, expansion volume, and the mass of the expanded layer. As shown, the expandable graphite density decreased from 114.38 to 11.02 kg/m^3^ (standard deviation: 34.43) when the expandable graphite content increased from 0 to 50 wt.%. This decrease in expandable graphite density is attributed to the significant increase in the expansion rate that was observed in case of combustion of a significantly smaller mass, which consequently resulted in a relatively low density. It is speculated that the interior of the expanded layer consists of a graphite layer with air occupying the surrounding environment.

### 3.3. Thermal Characteristics of Expandable Graphite Composites

**Figure 10:**
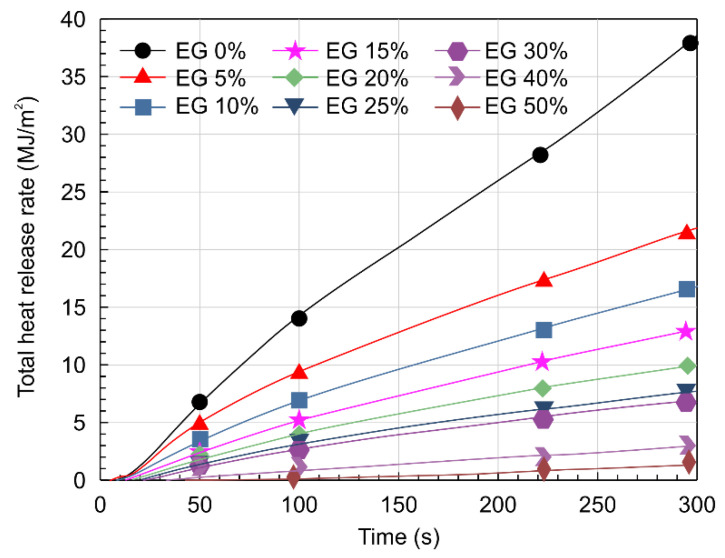
Variation in total heat released (THR) with combustion time.

Figure 10 presents the total heat released (THR) with the combustion time of the specimens. We observed that the THR decreased from 38.63 MJ/m^2^ to 21.94, 16.78, 13.14, 10.11, 7.76, 6.86, 3.80, and 2.50 MJ/m^2^ (standard deviation: 11.28) as the expandable graphite content increased from 0 to 50 wt.%. In addition, the ignition time varied from 13 to 66 s and was reduced with increasing expandable graphite content.

**Figure 11:**
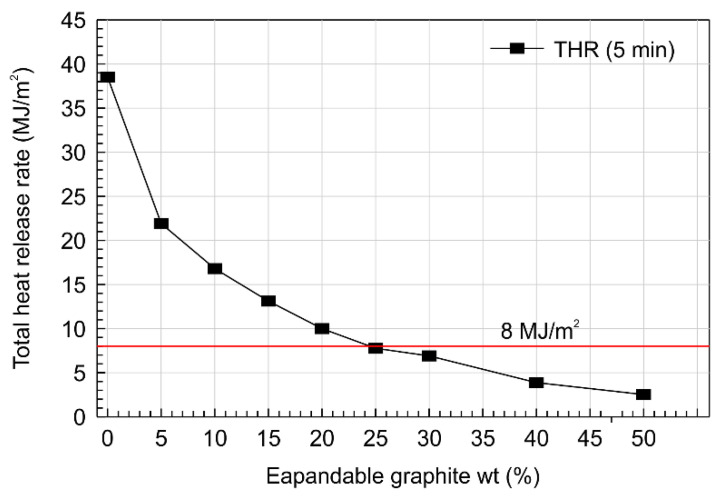
Variations in total heat released (THR) with expandable graphite content.

The THR as a function of the expandable graphite content is plotted in Figure 11 and compared to the performance standard of the flame retardance. A THR value of 6.86 MJ/m^2^ was obtained at an expandable graphite content of 30 wt.%. In addition, the corresponding THR was shown to fall below the threshold value of 8.0 MJ/m^2^, which was required to meet the performance standard for a flame retarding material in Table 3 below.

**Figure 12:**
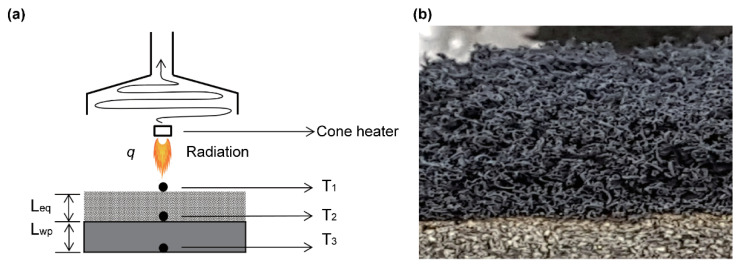
(**a**) Schematic of heat flow in cone calorimetry experiment and (**b**) image of expanded layer.

**Figure 13:**
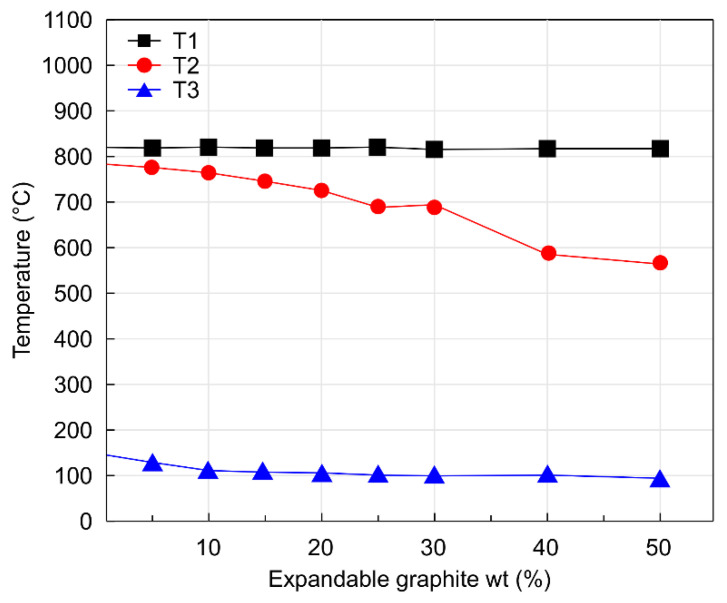
Temperature variations in the combustion experiment as a function of expandable graphite content.

Figure 12 depicts the schematic of the heat flow measured with thermocouples installed at the top and bottom of the specimen in the cone calorimetry experiment. The schematic shows the process of heat transfer from a cone heater (combustion temperature of 819 °C, T_1_) to the interface of the expanded graphite layer (L_eq_), and the unburned wood particle layer (L_wp_) (interface temperature T_2_), and finally to the bottom of the specimen (temperature T_3_). Figure 13 shows the temperature change at each position according to the expandable graphite content in the composite. As shown, T_1_ remained unchanged at 819 °C, whereas T_2_ decreased from 791.2 to 557.1 °C (standard deviation: 86.11), and T_3_ decreased from 121.1 to 98.7 °C as the expandable graphite content increased. The temperature change in the combustion layer where the combustion occurred (ΔT_12_) increased from 27.8 to 261.9 °C as the content of expandable graphite increased. On the other hand, the temperature change of the layer where combustion did not occur (ΔT_23_) decreased from 670.1 to 458.4 °C. Therefore, as the content of expandable graphite increased, the temperature T_2_ of the combustion layer gradually decreased. It seems that as the content of expandable graphite increases, the increase in the temperature difference (ΔT_12_) between the input combustion temperature and the temperature of the combustion layer increases the thickness of the expanded layer, which decreases the heat transfer rate.

The thermal conductivity was calculated separately for L_eq_ where combustion occurred, and for the unburned wood particle layer (L_wp_) of the specimen by the following equation:

(3) $$\mathrm{Qx}=-\mathrm{kA}\frac{\mathrm{dT}}{\mathrm{dx}},\;k=\frac{\mathrm{Qx}}{A}\cdot \frac{L}{\Delta T}=q\frac{L}{\Delta L}$$

where Qx is the total heat flow rate (W), k is the thermal conductivity (W/m·K), q is the heat flux (W/m^2^), L is the thickness of the layer (mm), A is the cross-section area (m^2^), and ΔT is the difference in temperature (K).

**Figure 14:**
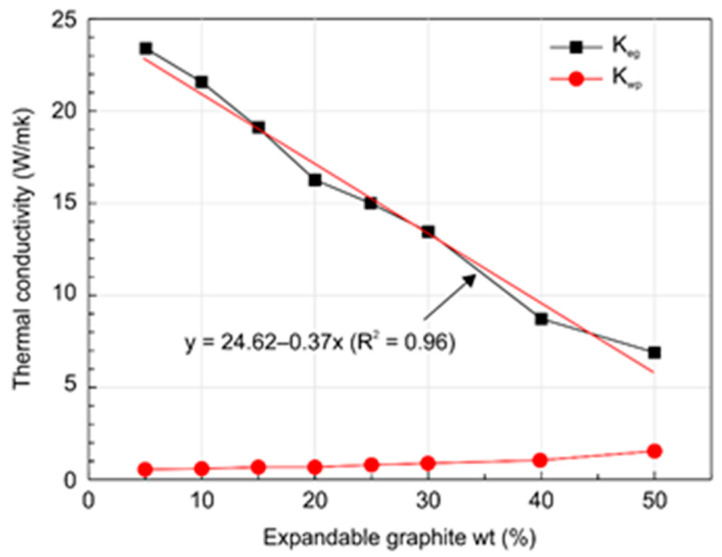
Thermal conductivity variations with expandable graphite content.

Figure 14 shows the variation of thermal conductivity in the expandable graphite content. As shown, the thermal conductivity of the expanded layer (k_eg_) decreased from 24.62 W/m·K at an expandable graphite content of 0 wt.%, to 7.83 W/m·K at expandable graphite content of 50 wt.% (standard deviation: 6.19). However, the thermal conductivity of the unburned wood particles specimen layer (k_wp_) increased from 0.40 W/m·K at 0 wt.% of expandable graphite content to 1.07 W/m·K at 50 wt.% of expandable graphite content (standard deviation: 0.23).

Consequently, the expanded layer (L_eg_) disturbs the heat transfer. Therefore, the temperature T_2_ at the bottom of the expanded layer decreases, along with k_eg_. However, the thermal conductivity of the unburned wood particle specimen layer (k_wp_) increases due to the higher thermal conductivity of the expandable graphite.

The thermal resistance was calculated based on Fourier’s Law, given as follows:

(4) $$R=\frac{\Delta T}{\mathrm{Qx}}=\frac{\Delta T}{A\cdot q}=\frac{\Delta T}{A\cdot k\cdot \frac{\Delta T}{L}}=\frac{L}{A\cdot k}$$

where R is the rate of thermal resistance (a property of the component (K/W)).

**Figure 15:**
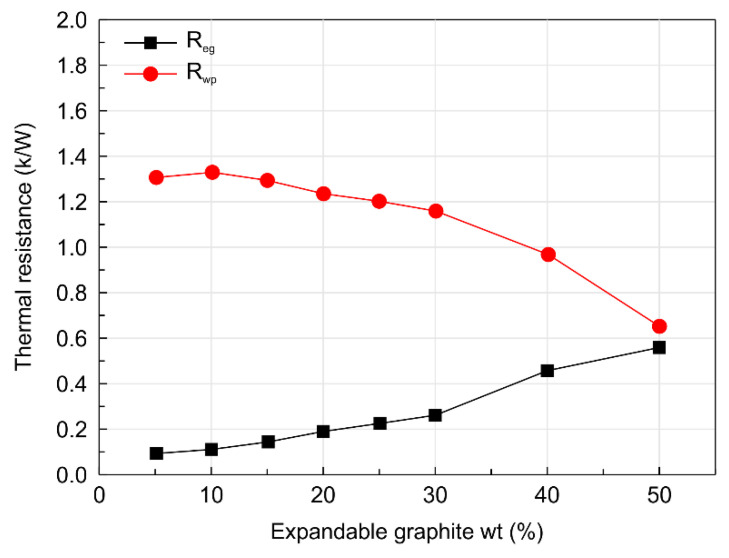
Thermal resistance variations with expandable graphite content.

The thermal resistance was calculated separately for the expanded layer and the non-combustive wood particle layer based on the calculation results of thermal conductivity. Figure 15 shows the variation in the thermal resistances of the expanded layer and the unburned wood particle layer of expandable graphite. We observed that, with the change in expandable graphite content from 0 to 50 wt.%, the thermal resistance of the expanded layer (R_eg_) increased from 0.05 to 0.64 K/W (standard deviation: 0.20), whereas the thermal resistance of the unburned wood particle layer (R_wp_) decreased from 1.35 to 0.80 K/W (standard deviation: 0.19).

In general, thermal conductivity tends to increase when expandable graphite exists in the unburnt state. However, when expandable graphite undergoes combustion, thermal conductivity decreases due to the expandability and thermal resistance of graphite that inhibit heat transfer.

**Figure 16:**
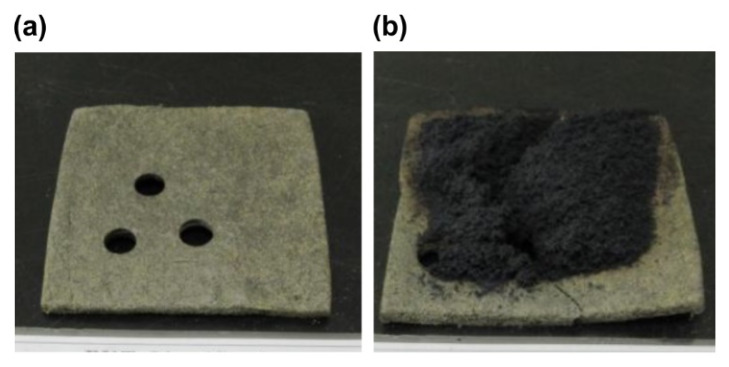
Images of (**a**) before and (**b**) after combustion for smoke toxicity test.

Figure 16a,b provide the images of specimens before and after the smoke toxicity tests. To determine the smoke toxicity, tests were performed twice in accordance with the standards for flame retardant performance.

For this test, we used a smoke toxicity tester specified in the flame retardancy test method for building interior materials and structures in KS F 2271(2006). Six samples with dimensions of 200 mm × 200 mm × 20 mm were prepared using an expandable graphite content of 0 wt.% (Case 1, 3 samples) and 30 wt.% (Case 7, 3 samples), as this condition resulted in a THR of 38.63, 6.86 MJ/m^2^ in the cone calorimetry combustion experiment and exhibited fire retardancy. After sample preparation, the specimen was left untouched for more than a month, dried in a desiccator at 35–45 °C for > 24 h, and then preserved for an additional 24 h. Tests were conducted in a well-controlled environment with a temperature of 20.0 ± 0.3 °C and humidity of 67% ± 2%.

Figure 16a is the image of a specimen with three perforated holes 25 mm in diameter, and Figure 16b shows its post-combustion state after the test.

Upon combustion, the time elapsed until a laboratory white mouse stopped its activity was used as the measure for the smoke toxicity test result. Table 4 shows the time to inactivity of the smoke toxicity test. The time to inactivity was 14.9 min on average and exceeded the toxicity standard for flame retardant performance (Table 3, time to inactivity > 9 min). In the case of wood particles (EG 0 wt.%), the average time to inactivity was 5.8 min, which does not meet the required standard. In the case where the expanded graphite was 30 wt.% of composite material, the time to inactivity apparently increased because the burned mass was smaller compared to that observed for wood particles, thus reducing the generated amount of harmful gases such as CO and CO_2_ [27].

Moreover, the ISO 1182, ISO 5660-1 standards for fire retardant material for evaluating flame retardation in case of the expandable graphite composite material are shown in Table 3. It was confirmed that the performance of the flame-retardant material was adequate for an expansion graphite content of 30 wt.% or more. The total heat release was 6.86 MJ/m^2^, while the time required to exceed a heat release rate of 200 kW/m^2^ was less than 10 s. No change was observed in the core material on properties such as melting, penetrating cracks, and holes. The standard time for the exhibition of gas toxicity was observed to be 9 min.

### 3.4. Changes in the Microstructure of Expandable Graphite

**Figure 17:**
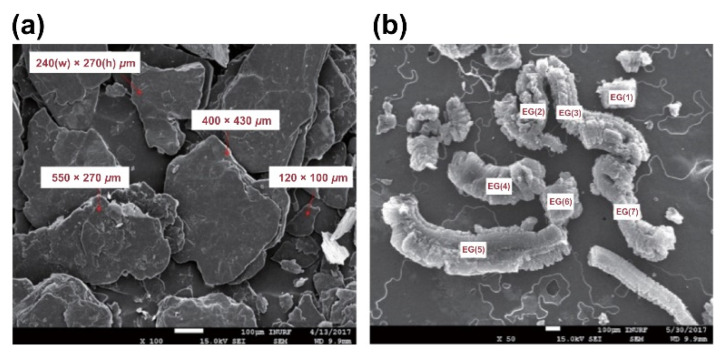
Images of expandable graphite at (**a**) 100× magnification and (**b**) 50× magnification.

The microstructure of expandable graphite before combustion and that of expanded graphite after combustion were analyzed using field emission scanning electron microscopy (FE-SEM). Figure 17a depicts the microstructure of the expandable graphite prior to its heating. The size of the expandable graphite before combustion was 120–550 μm in width, 100–430 μm in length, and approximately 10 μm in thickness, as determined from three microscopy measurements. The morphology was revealed to adopt a laminated layer structure, characterized as a plate type with flat surfaces. Figure 17b displays the morphology of the expanded graphite after combustion. Upon heating, expanded graphite experiences expansion in the interlayer spacing and thereby adopts a worm shape. The size of the expanded graphite was measured as 180–270 μm in width and 250–1470 μm in length. The thickness of the expanded graphite upon heating was shown to increase approximately 20 times compared to the initial thickness of 10 μm prior to its combustion.

**Figure 18:**
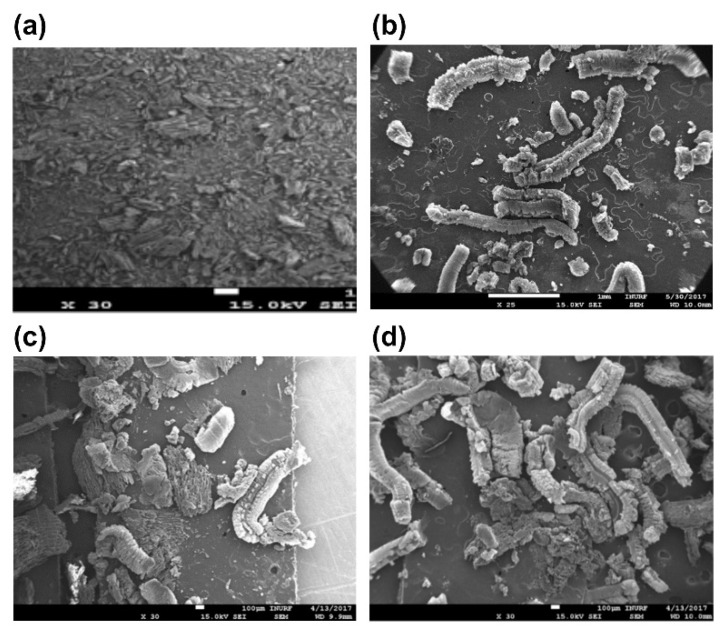
Fine image changes with expanded graphite wt.%: (**a**) 0, (**b**) 10, (**c**) 30, and (**d**) 50 (at 30× magnification).

Figure 18 presents the microstructures of expanded graphite composites with increasing content of expandable graphite after cone calorimeter combustion. Figure 18a shows the structure of a specimen of 100 wt.% wood particle without any added expandable graphite at a 30× magnification. The wood particles were converted to char of approximately 100–200 μm in size after combustion. Figure 18b–d displays the post-combustion images of composites with 10, 30, and 50 wt.% expandable graphite, respectively, at a 30× magnification. As the amount of expanded graphite was proportional to the content of added expandable graphite, it was confirmed that there was an increase of approximately 2 times.

**Figure 19:**
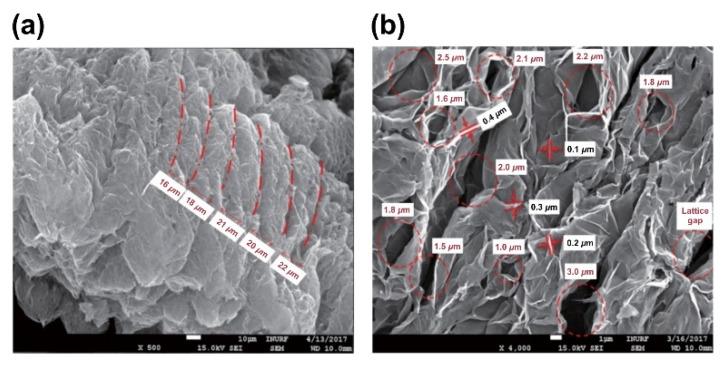
(**a**) Lattice layer gap in expanded graphite (500× image) and (**b**) ultra-fine film structure of expanded graphite (4000× image).

Figure 19a depicts the morphology of expanded graphite after heating. The magnified image of the microstructure revealed that the fine lattice layer repeated with 16–22 μm spacing. Figure 19b shows the image of the expanded graphite at a 4000× magnification. The thickness of the lattice layer was 0.1–0.4 μm with internal gaps of 1.0–2.5 μm. Upon exposure to heat, the expandable graphite expands. As the expandable graphite content increases, the thickness of the expanded graphite increases, and the distribution of the fine lattice layer also increases in proportion. Therefore, it can be deduced that the transfer of heat and gas contained in the region within the expanded graphite is inhibited due to the fine lattice layer structure. As a result of this phenomenon, the increase in expandable graphite content was observed to have a direct impact on the decrease in thermal conductivity of the composite.

## 4. Conclusions

In this study, a cone calorimetry experiment was conducted for composites with expandable graphite and wood particles. Moreover, their physical and thermal characteristics, smoke toxicity test results, and microstructures were analyzed. The study had the following conclusions:

- As a result of the DSC analysis, a faster heat absorption rate compared that of the pure expandable graphite was observed when the expandable graphite was added to the wood particles. This was due to the absorption of a large amount of heat for the carbonization of the wood particles in the specimen. In the XRD analysis after combustion, the diffraction peak level of the wood particles decreased, and carbon was detected by the combustion of the wood particles and thermal decomposition of expandable graphite.
- Analysis of the physical properties of the expandable graphite–wood particle composites revealed that increasing the expandable graphite content from 0 to 50 wt.% increased the expansion rate from 0% to 341.7%. Given that expandable graphite is a mixture of sulfuric acid and an oxidant and is produced from processes that involve oxidation, washing, drying, heating, and expansion, such an observation was shown to result from the wide expandability layer that arises when expandable graphite expands upon exposure to heat and produces gas.
- Analysis of the thermal characteristics of the expanded graphite layer (L_wp_) composites with added expandable graphite showed that increasing the expandable graphite content from 0 to 50 wt.% decreased the total heat released from 38.63 to 2.50 MJ/m^2^, and when expandable graphite contained more than 30%, it showed a stable fire retarding effect. It was concluded that this resulted from the delay in ignition time and decrease in the total heat released with increasing expandable graphite content.
- Increasing the expandable graphite content from 0 to 50 wt.% decreased the thermal conductivity of the expanded graphite layer from 24.62 to 7.83 W/m·K. In general, the thermal conductivity of expandable graphite tends to increase when it exists in the unburnt state. However, when expandable graphite undergoes combustion upon exposure to heat, both the expandability of graphite and the thickness of the expanded layerincrease, which in turn tends to decrease the thermal conductivity in the expanded layer due to the hindered transport of heat caused by the heat transfer shielding effect.
- Smoke toxicity tests with the 30 wt.% expandable graphite specimen showed that the time to inactivity of the white mouse was 14.9 min on average, which meets the lethal toxic potency standard (time to inactivity > 9 min) of a fire retardant. In the case of wood particles, the time to inactivity was 5.8 min, which does not meet the standard. In the case of the expandable graphite 30 wt.% composite material, the time to inactivity appears to have increased because of the smaller burned mass as compared to that in wood particles, thus reducing the generated amount of harmful gases such as CO and CO_2_.
- The structure of expandable graphite before combustion was characterized by a laminated layer structure 120–550 μm in width, 100–430 μm in length, and approximately 10 μm in thickness. The microstructure of the expanded graphite was revealed to be a worm shape, and the amount of expanded graphite was observed to increase in proportion with increase in the expandable graphite content. Additionally, the microstructure of expanded graphite was characterized by a fine lattice layer with a 16–22-μm interlayer spacing, repeating at a thickness of 0.1–0.4 μm and an interval of 1.0–2.5 μm, and the enhanced thermal resistance of the composite was due to the decrease in thermal conductivity.

In conclusion, we found that an increase in expandable graphite content results in an increased expansion rate and has a direct effect on the decrease in thermal conductivity due to the distributed expanded graphite and its fine lattice structure. In addition, the developed composite was found to satisfy the flame-retardant performance standards at an expandable graphite content of more than 30 wt.%. The results of this study are expected to serve as a foundation for early development and commercialization of expandable graphite–wood particle composites, which may contribute to minimizing civilian casualties and property damage caused by fires.

## Figures and Tables

**Table 1:** Compositions and morphologies of wood particles and expandable graphite.

Composition	Wood Particles	Expandable Graphite
Content (%)	Cellulose ≤ 50% Hemicellulose ≤ 25% Lignin ≤ 25% Chemical Content - Carbon: 55.61 wt.% - Oxygen: 44.39 wt.%	Fixed carbon: 93.8% Ash: 6.2% Moisture: 0.68% pH: 4.5 Expansion volume: 210 mL/g Expansion temperature: 180–200 °C
Size	80–100 mesh	80 mesh (82%)
Morphology	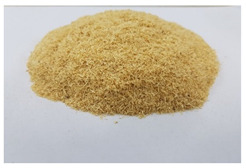	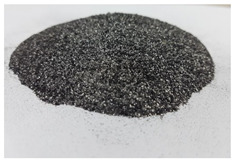

**Table 2:** Compositions of the expandable graphite composite materials.

Specimen		Mass of Composite Materials (g)			
Case	EG wt.%	Expandable Graphite	Wood Particles	Common Binder	Sum
Case 1	EG 0 wt.%	0	100	300	400
Case 2	EG 5 wt.%	21	100	300	421
Case 3	EG 10 wt.%	45	100	300	445
Case 4	EG 15 wt.%	71	100	300	471
Case 5	EG 20 wt.%	100	100	300	500
Case 6	EG 25 wt.%	134	100	300	534
Case 7	EG 30 wt.%	172	100	300	572
Case 8	EG 40 wt.%	267	100	300	667
Case 9	EG 50 wt.%	400	100	300	800

**Table 3:** ISO 1182, ISO 5660-1 standards for fire retardant material [26].

Item	Semi Noncombustible Material	Flame Retardant Material
THR (MJ/m^2^)	Below 8 MJ/m^2^	Below 8 MJ/m^2^
Time in which the heat release rate is exceeded 200 kW/m^2^ continuously	Below 10 s	Below 10 s
All core melt, penetrating cracks, slot change, etc.,	No core melt, penetrating cracks, slot change	No core melt, penetrating cracks, slot change
Toxicity	Experimental mouse Activity over 9 min	Experimental mouse Activity over 9 min
Test time	10 min (600 s)	5 min (300 s)

**Table 4:** Result of the time to inactivity of smoke toxicity test (min).

Specimen	EG 0 wt.% (Wood Particles 100%)	EG 30 wt.%
No.1	5.5	14.8
No.2	6.2	15.4
No.3	5.7	14.5
Average	5.8	14.9
